# Magnetic-Assisted Fractionation of Bone Marrow Cells into Subsets Differing in CD45 Expression Levels, Surface Phenotypes and Functional Properties

**DOI:** 10.3390/cells15171517

**Published:** 2026-08-23

**Authors:** Oleg F. Kandarakov, Natalia S. Polyakova, Alexander V. Belyavsky

**Affiliations:** Engelhardt Institute of Molecular Biology, Russian Academy of Sciences, Vavilov Str. 32, 119991 Moscow, Russia; oleg.kandarakov@gmail.com (O.F.K.); natae05@yandex.ru (N.S.P.)

**Keywords:** MACS, MACS-MEL, FACS, magnetic selection, bone marrow, CD45, CD11b, Gr-1, CD115, CD117, CD19, TER-119, CFUs

## Abstract

Cells of higher organisms express numerous cell surface proteins, and their spectrum and level of expression are directly related to cells’ functions. The technology of mass cell selection based on the surface protein expression levels may be highly important both for basic research and cell therapy applications. We have previously developed a method of magnetic selection of cells differing in surface marker expression levels, which we term here MACS-MEL (Magnetic-Assisted Cell Selection by Marker Expression Levels). The method demonstrated its effectiveness in the artificial model system, namely retrovirally transduced NIH 3T3 cells. However, whether it was also applicable to complex natural cell populations remained unclear. In the current study, we validated the MACS-MEL approach by separating mouse bone marrow (BM) cells into fractions according to the expression of pan-hematopoietic marker CD45. In the basic protocol, two-stage fractionation of CD45^+^ cells from BM was performed using selection of cells consecutively with 2 μL and 8 μL of anti-CD45 magnetic beads, resulting in isolation of CD45^high^ and CD45^int^ cell populations. To explore in full the potential of the method, the extended protocol was also tested, where a third selection stage with 30 μL of anti-CD45 beads was added. The isolated cell fractions were analyzed by flow cytometry for CD45 expression, as well for CD11b, Gr-1, CD117, CD115 and CD19 markers, while their in vitro progenitor function was assessed by quantitating colony-forming units (CFUs) in methyl cellulose. The results of analysis demonstrate that the isolated cell fractions significantly differed both in their surface phenotypes and CFU potential. In particular, cell fractions with progressively reduced CD45 expression were characterized by decreasing expression of myeloid differentiation markers CD11b and Gr-1, as well as B-lymphoid marker CD19. The expression of stem/progenitor cell marker CD117, on the contrary, significantly increased. The CFU frequency also strongly correlated with decrease in CD45 expression, while the differentiation potential of CFUs differed substantially in various cell fractions. In general, our results demonstrate that less differentiated hematopoietic cells in mouse BM studied using in vitro tests are characterized by lower CD45 expression levels, in full accordance with data obtained in human system. Successful validation of the MACS-MEL in a BM system, characterized by existence of multiple cell types and high phenotypic and functional heterogeneity, demonstrated the effectiveness, simplicity and affordability of this method. The MACS-MEL approach can be applied for mass selection of cells based on differential marker expression and may yield cell subsets suitable for advanced cell therapy applications.

## 1. Introduction

Cells of higher organisms express numerous cell surface proteins, and their repertoires are directly related to cells’ functions. The differences in surface marker expression reflect cell state and role in the body in normal and pathological conditions,= and have fundamental importance for biomedical research [1,2], as well as have use for diagnostic and therapeutic applications in medicine. This makes methods of surface marker-based cell separation virtually indispensable for these fields. Magnetic cell sorting approaches based on selection of surface markers provide certain advantages over fluorescence-activated cell sorting. Among them are the ability to isolate large numbers of cells required for clinical and certain basic applications, short processing time and minimization of negative effects on cells while maintaining cells’ original characteristics. Although magnetic selection is generally considered only suitable for isolation of populations either positive or negative for a selected marker, we previously developed an approach that allows using magnetic selection to fractionate cells into populations with high and low surface marker expression levels [3]. This method, which we term here MACS-MEL (Magnetic-Assisted Cell Selection by Marker Expression Levels), makes it possible to select cells based on the level of marker expression in a short time and at a minimal cost. The method demonstrated its effectiveness in the artificial model system, namely, NIH 3T3 cells transduced by bicistronic retroviral vectors expressing the surface protein LNGFR as a selection marker, as well as EGFP or DsRedExpress2 as indicators of the expression level [3].

However, whether the method was also applicable to complex natural cell populations remained unclear. In the current study, we validated the MACS-MEL approach in a non-model system by separating mouse bone marrow (BM) cells into fractions according to the expression of pan-hematopoietic marker CD45. BM is a tissue of primary importance for hematopoiesis and with a vast phenotypic cell diversity, including surface maker expression [4,5], and was thus used as the cellular system for validation of the MACS-MEL. CD45 receptor tyrosine phosphatase, a hallmark of all nucleated hematopoietic cells and an important regulator of signaling cascades in immune cells, was selected as a selective marker protein [6]. CD45 expression disorders often cause autoimmune, immunodeficiency, and oncological diseases [7]. It is therefore of considerable interest for biology and medicine, including its use as a target for biotechnological manipulations [8,9,10].

During mouse embryogenesis, CD45 expression begins in the hematopoietic cells of the yolk sac, while in the aorta-gonad-mesonephros region, CD45^low^c-kit^+^ cells demonstrate the greatest CFU activity in vitro [11]. In the adult humans, the level of CD45 expression increases with the maturation of cells of B and T lymphoid lines, as well as the monocytic cell line expressing CD11b and CD14 [12], while in umbilical cord blood, multipotent precursors are CD45-low [13]. Paradoxically, despite the almost ubiquitous presence of the CD45 marker in hematopoietic cells, the studies connecting the functional or differentiation state with the expression level of CD45 in the mouse system seem to be lacking.

An issue that is important for the experiments described in this study is the potential existence of CD45-negative hematopoietic progenitors. Indeed, CD45-negative erythroid and lymphoid progenitors have been discovered earlier in murine BM [14]. One should also mention the reported existence of CD45-negative cells in skeletal muscles that can reconstitute multilineage hematopoiesis in irradiated mice [15]. The issue, however, remains controversial as another study demonstrated these cells to be CD45-positive and migrate from BM [16].

Another relevant question is whether CD45 expression in BM is specific for hematopoietic cells, or if certain non-hematopoietic cells are also CD45-positive. There is currently a significant controversy as to whether mesenchymal stem/stromal cells (MSCs) in bone marrow express CD45 marker. Cultured MSCs are clearly CD45-negative, whereas some studies report that MSCs in BM are CD45-low or even CD45-intermediate [17,18], while others demonstrate that they are CD45-negative [19,20].

Summarizing the above, BM cells are an adequate and interesting object for testing the MACS-MEL approach in a natural system. Although validation of MACS-MEL in this study was carried out on the basis of CD45 expression, the isolated cell fractions also demonstrated substantial differences in surface phenotype, in particular expression of differentiation markers CD11b, Gr-1 and CD19, as well as CD117 (c-Kit), a known regulator of stem/progenitor cells [21,22]. Additionally, a colony forming unit (CFU) analysis of the isolated fractions in methylcellulose demonstrated significant differences in CFU frequencies that were negatively correlated with CD45 expression.

## 2. Materials and Methods

### 2.1. Isolation of Bone Marrow Cells

All animal procedures were performed in accordance with Russian regulations on animal protection and approved by the local Ethics Review Committee at EIMB RAS (Protocol No. 3 from 27 October 2022). BM cells were obtained from the femoral bones of the C57Bl/6 mouse using aseptic techniques. Cells were then suspended in 10 mL of DMEM-high glucose (#C420E, PanEco, Moscow, Russia), centrifuged for 10 min at 300× *g* and +4 °C, and the cell pellet was suspended in PBE (1x PBS, 0.5% newborn bovine serum (#IVD8002, Globe Kang, Qinhuangdao, China), 2 mM EDTA). The cell suspension was passed through a 40 µm cell strainer (#93040, SPL Life Sciences, Pocheon-si, Republic of Korea) and centrifuged for 10 min at 300× *g* and +4 °C, the cell pellet was suspended in PBE.

### 2.2. Two-Stage Magnetic Selection of CD45-Positive BM Cells (Basic Protocol)

CD45 MicroBeads (#130-052-301), hereinafter referred to as MBs, LS columns (#130-042-401) and a QuadroMACS magnet (#130-090-976) (all from Miltenyi Biotec, Bergisch Gladbach, Germany), were used for magnetic selection of CD45-positive cells from BM. Two-stage magnetic cell selection was performed under the laminar hood according to the manufacturer’s recommendations. In the first stage, 12 × 10^6^ BM cells were suspended in 98 µL of PBE, and 2 µL of MBs was added, mixed, and incubated for 15 min at +4 °C. After incubation, the unbound MBs were removed as follows: 2 mL of PBE was added to the cell suspension, and cells were centrifuged for 10 min at 300× *g* and +4 °C and resuspended in 500 µL of PBE. Thereafter, the cell suspension was loaded onto LS column that was mounted on the QuadroMACS and washed with 3 mL of cold PBE prior to cell application. The column flow-through was collected in a centrifuge tube, followed by column washes with 3 × 3 mL of cold PBE into the same tube (combined flow-through fraction 1). To recover the CD45-positive cells (column fraction 1), the column was removed from the QuadroMACS and mounted on a 15 mL tube, 5 mL of PBE was added to the column, and the suspension of detached cells was pushed through the column with a piston into the tube. The collected cells of the flow-through fraction 1 and column fraction 1 were centrifuged at 300× *g* for 10 min at +4 °C and suspended in 1–2 mL of PBE.

For the 2nd stage of selection, 10 × 10^6^ cells of the flow-through 1 fraction were centrifuged at 300× *g* for 10 min at +4 °C and resuspended in 92 µL of PBE. A volume of 8 µL MBs was then added to suspension, and the procedure described for the 1st stage of selection was repeated. The isolated CD45-positive cells of the first and second stages of selection were used for cytometry and quantitation of CFUs in methylcellulose. The cells in fractions were counted using a hemacytometer. Cell viability was determined using Trypan blue, as well as propidium iodide staining using flow cytometry.

For the extended protocol, isolation stages 1 and 2 were scaled up 2-fold, and the third selection stage was added using 6.4 × 10^6^ cells of the flow-through 2 fraction and 30 µL MBs.

### 2.3. Flow Cytometry

The following antibodies were used for surface phenotype analysis by flow cytometry: anti-mouse CD45-FITC (#103108, clone 30-F11), anti-mouse CD117-PE (#105807. clone 2B8), anti-mouse CD115-Brilliant Violet 421 (#135513, clone AFS98), anti-mouse/human CD11b-APC (# 101211, clone M1/70), anti-mouse Gr-1-FITC (#108405, clone RB6-8C5), anti-mouse CD45-APC (# 103112, clone 30-F11), anti-mouse CD45-Brilliant Violet 421 (#103134, clone 30-F11), anti-mouse CD19-APC (#115511, clone 6D5), TER-119-biotin (#133307, clone TER-119) and Streptavidin-PE (#405203) (all from BioLegend, San Diego, CA, USA). For cell staining, antibody cocktails were prepared by combining the antibodies with different fluorochromes.

The cells in selected fractions were centrifuged for 5 min at +4 °C at 300× *g*, resuspended in 100 µL of PBE, antibody mixes were added, and cell samples incubated in the dark at +4 °C for 30 min. After staining, the cells were washed from the antibodies with 2 mL of PBE buffer and stored at +4 °C for no more than 30 min before cytometry. To assess the viability of the selected populations, antibody-free cells were stained with Propidium iodide (10 µg/mL) for 1 min immediately before cytometry. Cell viability during selection was at least 90%.

For FACS selection, BM cells were labeled with a mixture of CD45-BV421, CD117-PE, and CD11b-FITC antibodies. CD45+ cells were then sorted using a BD FACSAriaIII Cell Sorter (BD Life Sciences, Franklin Lakes, NJ, USA) equipped with 405 nm laser and 450 ± 40 nm filter using 100 μm NOZZLE, and the purity mode Yield. Cells were sorted into PBS buffer (PanEco, Moscow, Russia) containing 5% FBS, 2 mM EDTA, 50 U/mL Penicillin and 50 µg/mL Streptomycin (PanEco), 2.5 µg/mL Amphotericin B (Biowest SAS, Nuaillé, France). BM cells and selected fractions were used for phenotype analysis by flow cytometry as described below, as well as for CFU analysis.

The cells were analyzed on a BDLSR Fortessa cytometer (BD Life Sciences) equipped with a 405 nm laser with 450 ± 25 nm filter, 488 nm laser with 530 ± 15 nm and 710 ± 25 nm filters, 561 nm laser with 586 ± 7.5 nm filter, and 640 nm laser with 670 ± 7 nm filter. Fluorescence of antibody fluorochromes was compensated using BD FACSDiva software, version 8.0.1. Microparticles of 10 μm (#72986), 20 μm (#74491) and 30 μm (#84135) size (Sigma-Aldrich Merck, Darmstadt, Germany) and particles of 6 μm size (#1451086 Proline Universal Calibration beads), Bio-Rad Laboratories Inc., Hercules, CA, USA) were used for gating BM cell populations. Cell debris and clusters were eliminated from the analysis by gating according to FSC-A/SSC-A and FSC-H/FSC-A, respectively. When measuring all samples, the cytometer settings were kept constant, and the number of collected events was at least 10,000. The cytometric data were processed using software Flowing Software, version 2.5.1 (https://bioscience.fi/services/cell-imaging/flowing-software; accessed on 25 May 2026 ). The MFI values were calculated using the same software.

### 2.4. CFU Analysis

To analyze the frequencies of CFUs in the isolated cell fractions of two-stage protocol, 3 replicates of 1 × 10^4^ cells/well were seeded into the wells of a 12-well plate containing MethoCult™ GF M3434 (# 03434, StemCell Technologies, Vancouver, BC, Canada). For the extended 3-stage protocol, due to the vast differences in CFU frequency, the different numbers of cells per well were seeded in three replicates for each fraction to obtain optimal numbers of colonies for counting. For the fractions column 1, column 2, flow-through 2, column 3 and flow-through 3, the seeded cell numbers were 3 × 10^4^ cells, 1 × 10^4^ cells, 1 × 10^4^ cells, 0.25 × 10^4^ cells and 10 × 10^4^ cells, respectively, per well.

In FACS experiments, BM cells and cells of CD45+ fractions were cultured in methylcellulose with the addition of 50 U/mL Penicillin and 50 µg/mL Streptomycin (PanEco, Russia) and 1 µg/mL Amphotericin B (Biowest SAS, Nuaille, France). The number of cells seeded per well for FACS fractions CD45^low^, CD45^int^ and CD45^high^ was 5 × 10^3^ cells, 15 × 10^3^ cells and 30 × 10^3^ cells, respectively.

The cells were cultured for 10–12 days at 37 °C and 5% CO_2_, the grown colonies were then counted and identified in accordance with the manufacturer’s Technical Manual “Mouse Colony-Forming Unit (CFU) Assays Using MethoCult™” (https://www.stemcell.com/mouse-colony-forming-unit-cfu-assays-using-methocult.html; accessed on 25 May 2026). The colonies were counted in three replicas for each variant. Colonies were photographed with an EVOS FL microscope camera (Thermo Fisher Scientific, Waltham, MA, USA).

CD45-normalized CFU frequency was calculated as follows:

CFU_CD45+_ = (100,000 cells × CFU)/(N × R_CD45+_)

where:

R_CD45+_—proportion of CD45+ cells in the fraction.

N—Number of cells seeded into the MethoCult™ medium.

CD117-normalized CFU frequency was calculated in a similar way.

### 2.5. Statistical Analysis

For the basic 2-stage selection protocol, 4 independent experiments were performed using one mouse per experiment. The data are presented, where appropriate, as mean ± standard deviation. Statistical values were calculated using Statgraphics Centurion Version 19.1.1 and online resource https://www.socscistatistics.com/; accessed on 25 May 2026.

## 3. Results

### 3.1. Basic Protocol: Two-Stage Magnetic Selection of BM Cells

**Figure 1:**
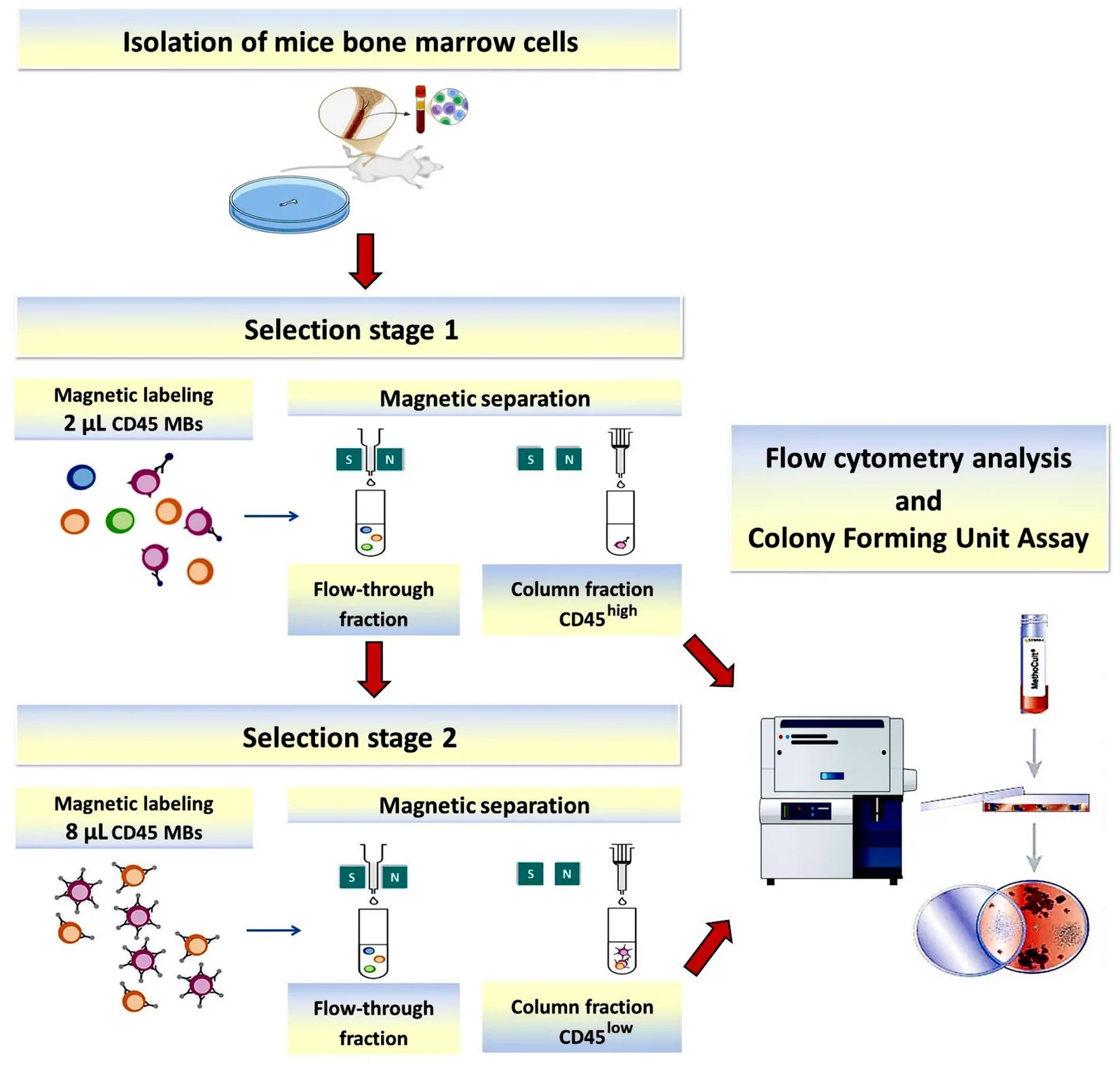
Experimental scheme (basic protocol).

To validate the MACS-MEL approach, the two-stage magnetic selection of BM was performed as described in the Materials and Methods. Both the initial BM cells and the isolated CD45-positive cell fractions were stained for the CD45 marker, myeloid markers CD11b, Gr-1, CD115, and the early marker CD117 (c-kit) and characterized by flow cytometry to trace the dynamics of changes in their expression during selection. The basic experimental protocol is depicted in the Figure 1. For the first selection round, BM cells were incubated with 2 µL MBs and separated on a column into two fractions, column fraction 1 and flow-through fraction 1. In the second selection round, the flow-through fraction 1 was incubated with 8 µL MBs and separated again into two fractions, column fraction 2 and flow-through fraction 2. The first and second round column fractions were analyzed by flow cytometry and for colony-forming activity by CFU assay in methylcellulose.

**Figure 2:**
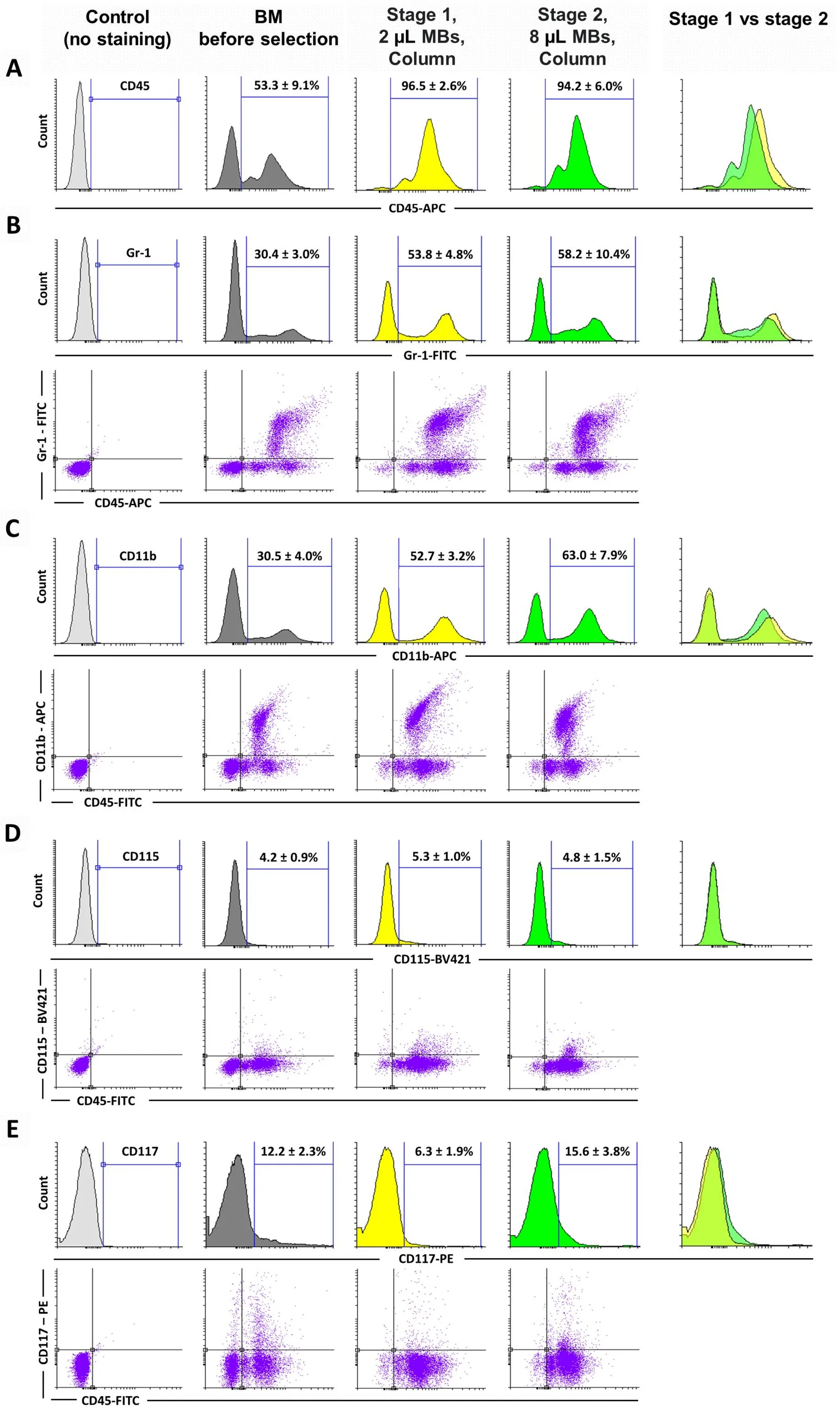
Flow cytometry analysis of cell fractions selected in the basic two-stage protocol. The gating strategy is described in Supplementary Materials. (**A**) CD45-APC, (**B**) Gr-1-FITC and Gr-1-FITC/CD45-APC, (**C**) CD11b-APC and CD11b-APC/CD45-FITC, (**D**) CD115-BV421 and CD115-BV421/CD45-FITC and (**E**) CD117-PE and CD117-PE/CD45-FITC. Control—unstained cells. The cytometric profiles are shown as one-dimensional histograms, as well as two-dimensional histograms vs. CD45 where appropriate. The profiles are representative of 1 of 4 replicate experiments. The numbers indicate percentage of positive cells; shown are mean values ± SD for 4 independent experiments.

Four independent experiments with the two-stage selection protocol were performed and demonstrated reproducible results; Figure 2 depicts the cytometric diagrams of one representative experiment of the four. The results of antibody staining of BM cells before and after selection are shown as one-dimensional histograms of CD45, as well as CD11b, Gr-1, CD115, CD117 markers with superimposed distributions of stained cells after stages 1 and 2 of selection, and as two-dimensional dot plot diagrams showing CD45 co-expression with the above hematopoietic markers (Figure 2).

**Figure 3:**
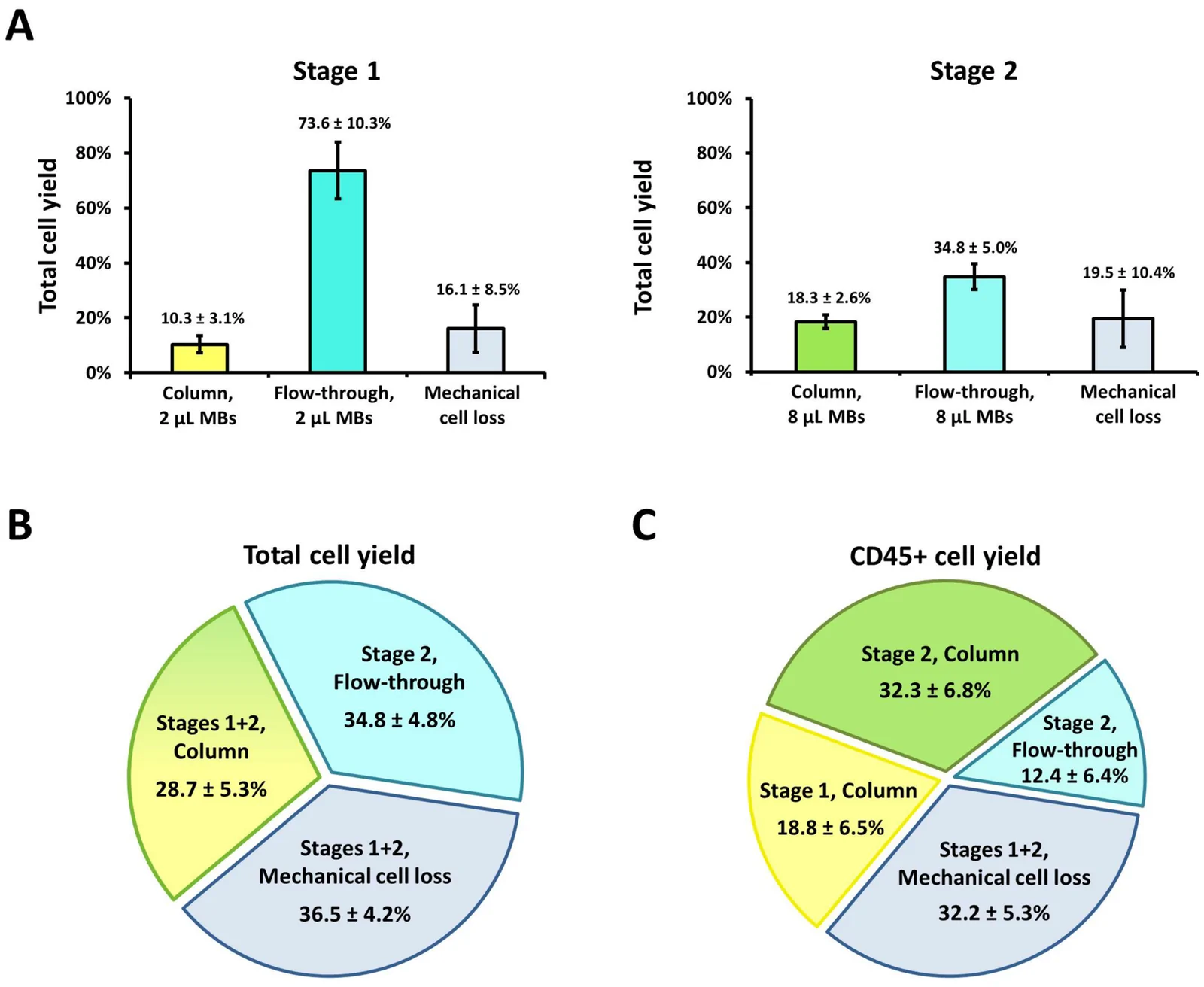
Parameters of cell populations during selection. (**A**) The yields of column and flow-through fractions in selection stages 1 and 2. (**B**) Cumulative yield of total cells after two selection stages. (**C**) Cumulative yield of CD45-positive cells after two selection stages. Shown are mean values ± SD for 4 independent experiments.

Note that only CD45^+^ BM cells were directly selected in these experiments, and as a result, the cells isolated in rounds 1 and 2 of selection differed in CD45 expression profile both from the initial population of BM cells and from each other (Figure 2A). In addition, the MFI of CD45^+^ cells in round 1 was 1.4 times higher than that of CD45^+^ cells in round 2 (Table 1). Thus, cells with higher CD45 expression (10,32% of total BM cells and 18.8% of CD45+ BM cells) were selected in the first round, while cells with lower CD45 expression (18.33% of total BM cells and 32.3% of CD45+ cells) were isolated in the second round (Figure 3C). We designate these cell populations as CD45^high^ and CD45^int^, respectively, for distinction in further analysis, although these terms may not fully correspond to those regularly used in flow cytometry due to principal differences in separation technologies.

### 3.2. Basic Protocol: Immunophenotypic Study of the 1st and 2nd Stage Cell Fractions

To characterize the isolated fractions of CD45-positive cells in more detail, we analyzed expression of CD117, CD115, CD11b and Gr-1 in them. The relevant results are shown in Figure 2B–E and Table 1, Table 2 and Table 3. The important markers of myeloid differentiation Gr-1 and CD11b demonstrated similar behavior in two fractions. Before selection, the proportion of Gr-1- and CD11b-positive cells in BM was about 30%; after selection, the proportion in the selected populations increased to about 54% and 58% in column fractions 1 and 2 for Gr-1^+^ cells, and to about 53% and 63% in column fractions 1 and 2 for CD11b^+^ cells. The differences between BM and fractions 1 and 2 for these markers were statistically significant (Table 1). Additionally, cells isolated in the 1st stage had substantially higher levels of Gr-1 and CD11b^+^ expression compared to the initial BM sample and cells of the 2nd stage, as judged by the average MFI levels (Table 2). Moreover, measured MFI levels for both markers were invariably higher for stage 1 compared to stage 2 cells in each of the four experiments. Nevertheless, Gr-1 the *p* value exceeded 0.05, indicating insufficient statistical significance at this number of measurements (Table 3).

CD115 (M-CSF receptor) is an important marker of myeloid differentiation of BM cells and plays a key role in the maturation of monocytes and their differentiation to macrophages. The proportion of CD115-positive cells in BM and in both selected fractions did not differ significantly (Figure 2D). The MFI of CD115^+^ cells was also similar in the 1st and 2nd stages and BM (Table 2 and Table 3). At the same time, CD117/c-kit (receptor of stem cell factor, an important regulator of stem/progenitor cells) demonstrated a contrasting behavior. The proportion of CD117-positive cells in BM was about 12%. In the CD45^high^ cell population selected in the 1st stage, the proportion of CD117^+^ cells dropped to 6%, whereas in the CD45^int^ population of the 2nd stage, this proportion increased above the BM levels to 15.6% (Figure 2E, Table 2 and Table 3). These results may point to a tendency for selection of less differentiated cells at the 2nd stage of selection.

Summarizing the expression patterns of differentiation markers, the levels of early and late myeloid differentiation markers correlated with the levels of CD45 in cell fractions isolated in the 1st and 2nd stages of selection. In the population selected in the 1st stage, a large decrease in CD117^+^ cell expression was observed together with higher expression levels of CD45 and late differentiation markers Gr-1 and CD11b, whereas in the 2nd round of selection, the expression patterns showed the opposite dynamics. Thus, the levels of CD117, CD11b and Gr-1 reflect the phenotypic differences between CD45^high^ and CD45^int^ cell subsets, with a potential impact on the CFU numbers in selected populations.

### 3.3. Basic Protocol: CFU Analysis of the 1st and 2nd Stage Cell Fractions

**Figure 4:**
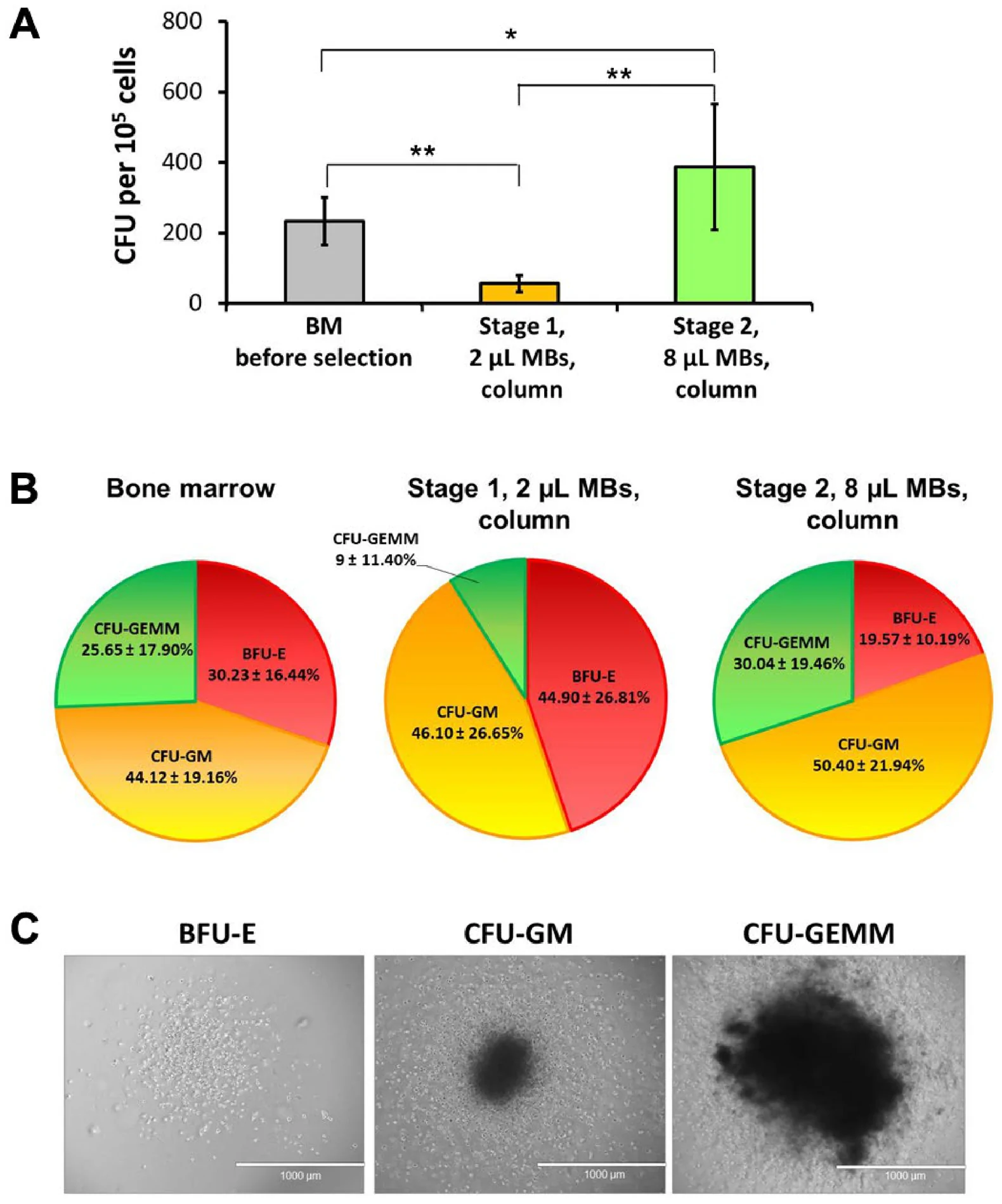
Analysis of CFU activity in MethoCult™ medium of CD45^high^ and CD45^int^ fractions selected in the basic 2-stage protocol. (**A**) The frequency of CFUs in BM and cell populations isolated in stages 1 and 2. The *p*-values were calculated using two-tailed Student’s *t*-test: ** *p* < 1 × 10^−4^, * *p* < 0.02. (**B**) The proportion of colony-forming units BFU-E, CFU-GM and CFU-GEMM in BM and selected populations, obtained by averaging CFU proportions in individual measurements. (**C**) The morphology of BFU-E, CFU-GM and CFU-GEMM colonies. Data represent 4 independent experiments, with measurements performed in triplicates. Shown are mean values ± SD for 4 independent experiments. Bar—1 mm.

To study the in vitro hematopoietic colony forming potential in selected populations, cells were seeded in MethoCult™ GF M3434 medium and colonies were counted 10–12 days thereafter. The results reveal significant differences in CFU frequency between selected fractions 1, 2 and BM. In particular, the average CFU frequency in the selected CD45^high^ cells was 7-fold and 4-fold lower than in CD45^int^ cells and BM cells, respectively (Figure 4A and Table 4), while the CFU frequency of the CD45^int^ population was 1.7-fold higher than in BM cells. Despite the substantial variations in bulk CFU frequencies in four experiments, the differences in total CFU frequencies between the fractions and BM were significant, as illustrated by *p* values in the Table 5.

Apart from the total CFU frequencies, substantial interest represents the comparison of isolated fractions by CFU type. Table 4 shows the frequencies of BFU-E, CFU-GM and CFU-GEMM colonies averaged for all four experiments. The pairwise comparison of these frequencies in CD45^high^ and CD45^int^ fractions (Table 5) demonstrate existence of significant differences between these fractions.

Importantly, the proportion of CFU-GEMM CFUs (the earliest progenitors with the ability to differentiate in vitro into granulocytes, erythrocytes, macrophages and megakaryocytes) in the CD45^int^ cell fraction was more than three times higher than in CD45^high^ fraction, while in the latter, about 45% CFUs were of BFU-E (erythroid lineage) compared to about 20% in the CD45^int^ (Figure 4B). The average proportion of CFU-GMs (granulocyte and macrophage progenitors) was rather similar in both isolated fractions and in BM. Thus, CD45^int^ fraction is apparently enriched in earlier progenitors with multiple in vitro differentiation potential.

The above results demonstrate clearly that with the two-stage magnetic selection, bone marrow cells can be fractionated according to the level of CD45 expression, resulting in the selection of the CD45^high^ population in the first stage and CD45^int^ population in the second stage. These populations differ substantially both in cell surface phenotypes and functional properties, primarily in the frequency of progenitor cells with in vitro differentiation capacity, as well as their differentiation potentials.

### 3.4. Extended Protocol: Immunophenotypic Study of CD45^high^ and CD45^low^ Cell Populations

Although the results described above demonstrated successful application of MACS-MEL protocol for CD45-expressing cells of mouse BM and thus presented the adequate validation of our approach, three essential considerations prompted us to perform an additional extended experiment consisting of three selection stages. First, in the basic two-stage experimental scheme, a substantial fraction of CD45-positive cells (about 12% of input CD45^+^ cells as in Figure 3C, and ca. 24% of combined stage 1 + 2 CD45^+^ cells) remained unselected and non-characterized. Second, the column 1 and column 2 fractions demonstrated a clear tendency for increase in CD117 positivity and CFU frequency in a cell fraction with reduced CD45 expression. It was therefore important to test whether this tendency persists also in cell fractions with even lower CD45 expression levels. Third, we were interested to check whether the reduction of CD45 levels on BM cells was accompanied by the decrease in expression of not only myeloid, but also lymphoid differentiation markers.

**Figure 5:**
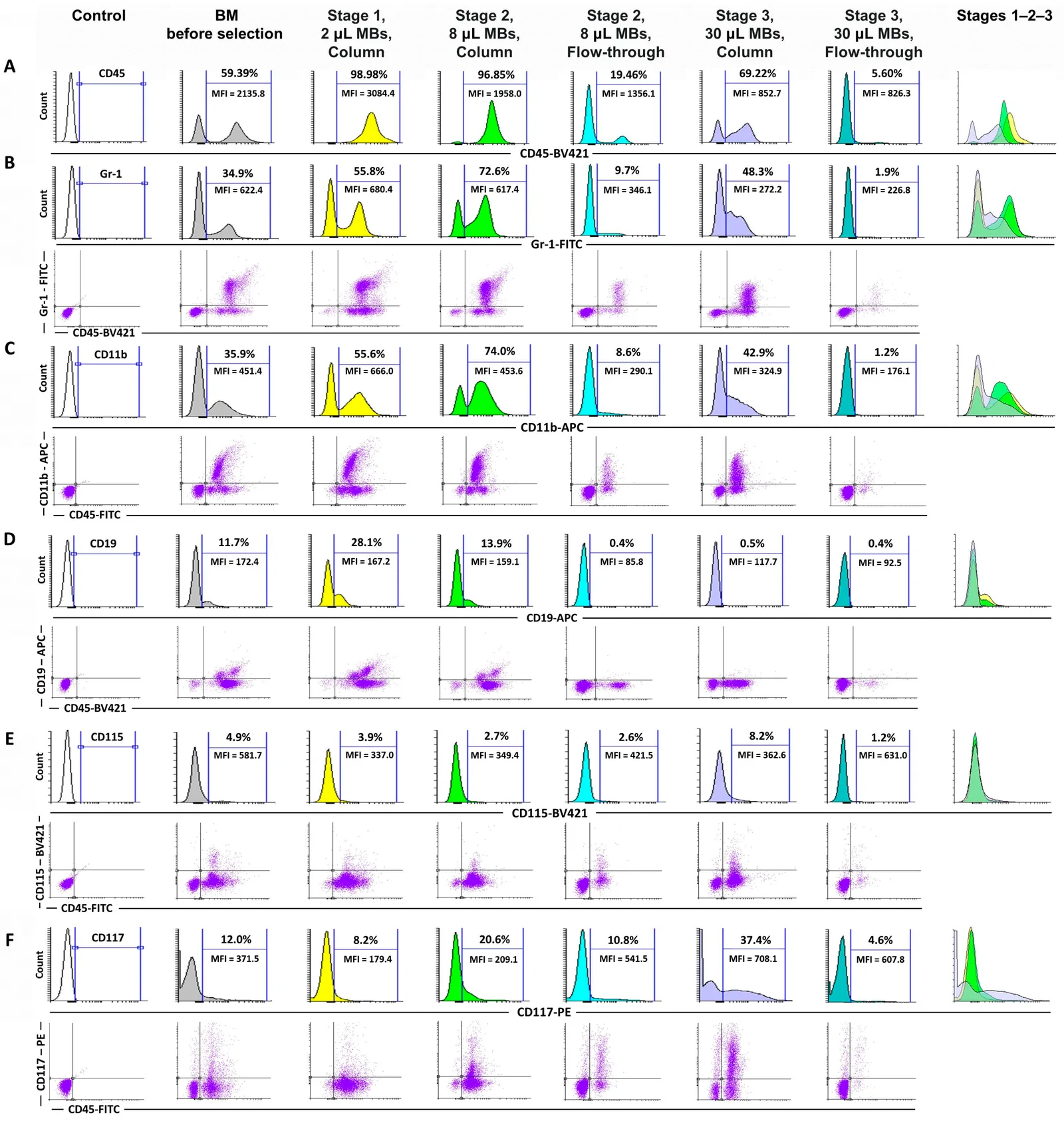
Flow cytometry analysis of cell fractions selected in the extended 3-stage protocol. (**A**) CD45-BV421, (**B**) Gr-1-FITC and Gr-1-FITC/CD45-BV421, (**C**) CD11b-APC and CD11b-APC/CD45-FITC, (**D**) CD19-APC and CD19-APC/CD45-BV421, (**E**) CD115-BV421 and CD115-BV421/CD45-FITC. (**F**) CD117-PE and CD117-PE/CD45-FITC. Control—unstained cells. The cytometric profiles are shown as one-dimensional histograms, as well as two-dimensional histograms vs. CD45 where appropriate.

We therefore performed an extended experiment where two selection rounds (scaled up 2-fold) were identical to those of basic protocol, whereas the third round was performed with 30 μL of MBs using 6.4 × 10^6^ cells of the column 2 flow-through fraction. Accordingly, the cytometric and CFU analyses were performed with three additional fractions, namely, flow-through 2, column 3 and flow-through 3. The data on cell yields at each stage of MACS-MEL are provided in Table 6, while the results of cytometric analysis are depicted in Figure 5.

According to cytometry diagrams of CD45 marker (Figure 5A), a majority of cells in the column 3 fraction is represented by CD45^low^ subset with MFI about 853 as compared to CD45^high^ and CD45^int^ subsets with MFIs 3084 and 1958 (column fractions 1 and 2, respectively). This indicates that a major part of cells with high and intermediate CD45 expression was removed from the population during selection stages 1 and 2. Analysis of Gr-1 and CD11b markers (Figure 5B,C) demonstrates that, while column 1 and 2 fractions contain cells with high and intermediate expression of these markers, fractions column 3 and flow-through 2 contain cells with mostly low, and to some extent, intermediate expression levels, as reflected in their MFI levels.

A highly interesting pattern was observed for the B-lymphoid marker CD19 (Figure 5D). In BM and both column fractions 1 and 2, two main populations of CD45^+^CD19^+^ cells were observed. However, CD19^+^ cells were nearly completely absent in the flow-through 2 and column 3 fractions, indicating that differentiated B-lymphoid cells, but possibly not the earliest lymphoid progenitors and B-committed precursors [23], were removed in the first two rounds of selection.

For the CD117/c-kit marker (Figure 5F), the consecutive column 1 to column 3 fractions demonstrated steady and significant increase in CD117 positivity, in particular ratio of CD117-positive to CD117-negative cells within the CD45-positive population. This ratio was about 0.09 for column 1, 0.26 for column 2, and 1.06 for column 3 fractions. This tendency was also conserved for flow-through 2 and flow-through 3 fractions (1.34 and 1.95 ratios, respectively). In addition, MFI value for CD117-positive cells demonstrated a similar tendency, raising from ca. 180 for column 1 to 708 for column 3 fractions, respectively. These observations clearly demonstrate that expression of an important stem/progenitor regulator CD117/c-kit is increased in parallel with a decrease in CD45 expression on BM cells. In contrast to the above hematopoietic markers, cytometric profiles for the myeloid regulator CD115 (M-CSF receptor) (Figure 5E) were fairly similar for all three column fractions, although a higher proportion of CD115 cells was observed in the column 3 fraction.

### 3.5. Extended Protocol: CFU Analysis of Stage 1–3 Fractions

**Figure 6:**
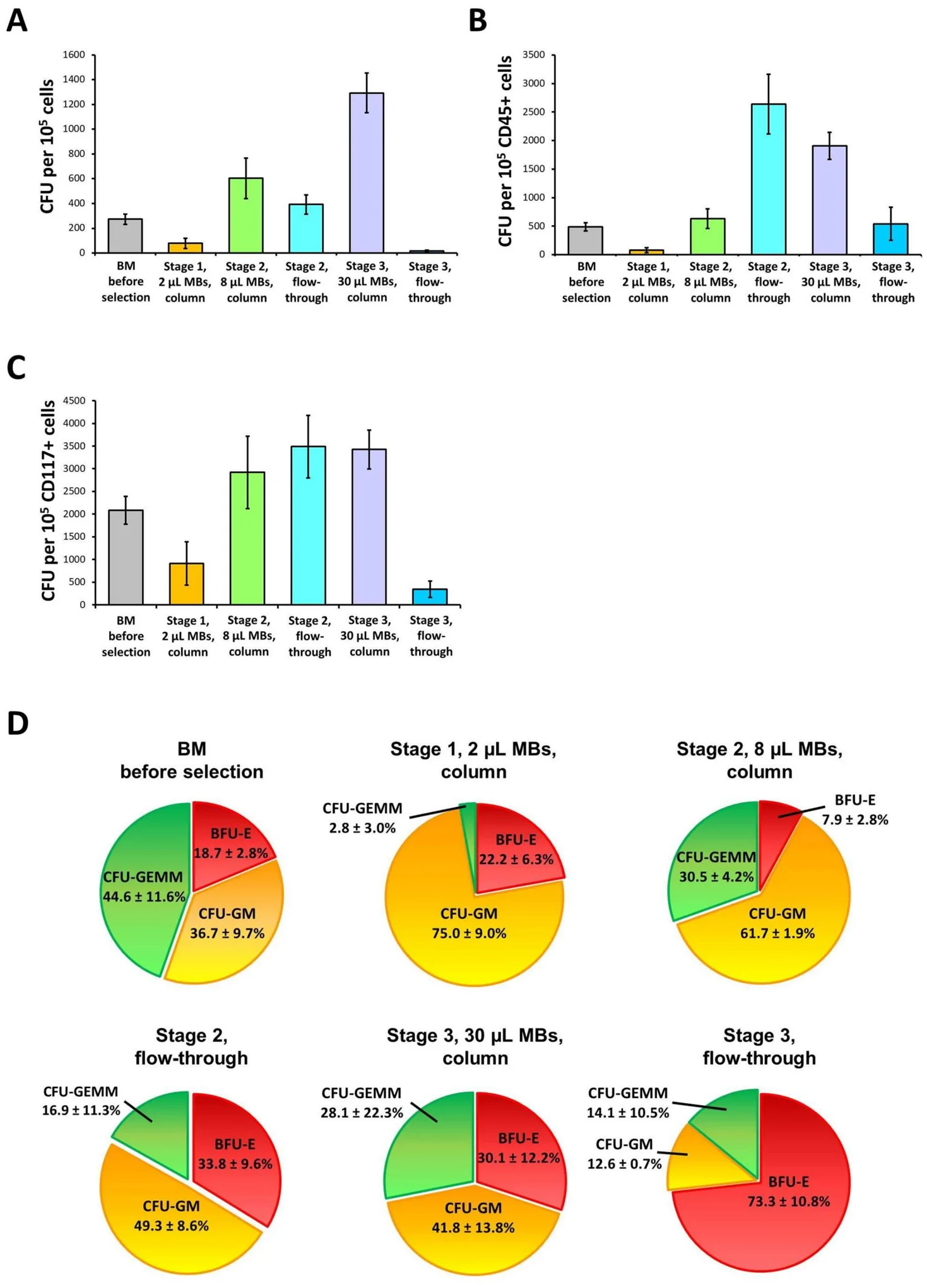
Analysis of CFU activity in MethoCult™ medium of cell fractions selected in the extended protocol. (**A**) CFU frequencies of selected fractions, (**B**) CD45-normalized CFU frequencies, (**C**) CD117-normalized CFU frequencies, (**D**) Proportions of colony-forming units BFU-E, CFU-GM and CFU-GEMM in populations. Measurements were performed in triplicates. Shown are mean values ± SD. CD45- and CD117-normalization procedures are described in the Section 2.4.

Analysis of CFUs in various fractions of extended protocol (Figure 6A) demonstrated that the frequency of CFUs was 273 per 10^5^ cells in BM, dropped to 79 in column 1 fraction, while increasing to 603 and 1293 per 10^5^ total cells for column 2 and column 3 fractions, respectively. Thus, the CFU frequency increased in successive isolation stages in parallel with reduction of CD45 expression.

For the flow-through fractions 2 and 3, the CFU frequencies were 393 and 16 per 10^5^ cells, respectively. However, the CD45-positive cells in fractions flow-through 2 and 3 were diluted by large numbers of non-hematopoietic CD45-negative cells unable to produce hematopoietic colonies. To obtain a more objective analysis of CFU activity for various fractions, we calculated the CFU frequency for CD45-positive cells only (Figure 6B). In this case, the frequency of CFUs was highest in the column 3 and flow-through 2 fractions, while for BM, as well as column 2 and flow-through 3 fractions these frequencies were fairly similar.

The rise of CFU frequency in consecutive column fractions was paralleled by the increase in CD117 levels, suggesting that colony-forming activity was dependent in part on CD117 expression. We therefore calculated the CFU frequencies per CD117-positive cells. The results of this analysis (Figure 6C) demonstrate that, indeed, these values were significantly less variable for BM and three consecutive fractions column 2, flow-through 2 and column 3, as compared to CFU frequencies per CD45-positive cells. These data agree with the assumption that CD117 expression level in cell subsets is an important factor contributing significantly to their CFU activity.

### 3.6. FACS Selection of CD45+ BM Cells

**Figure 7:**
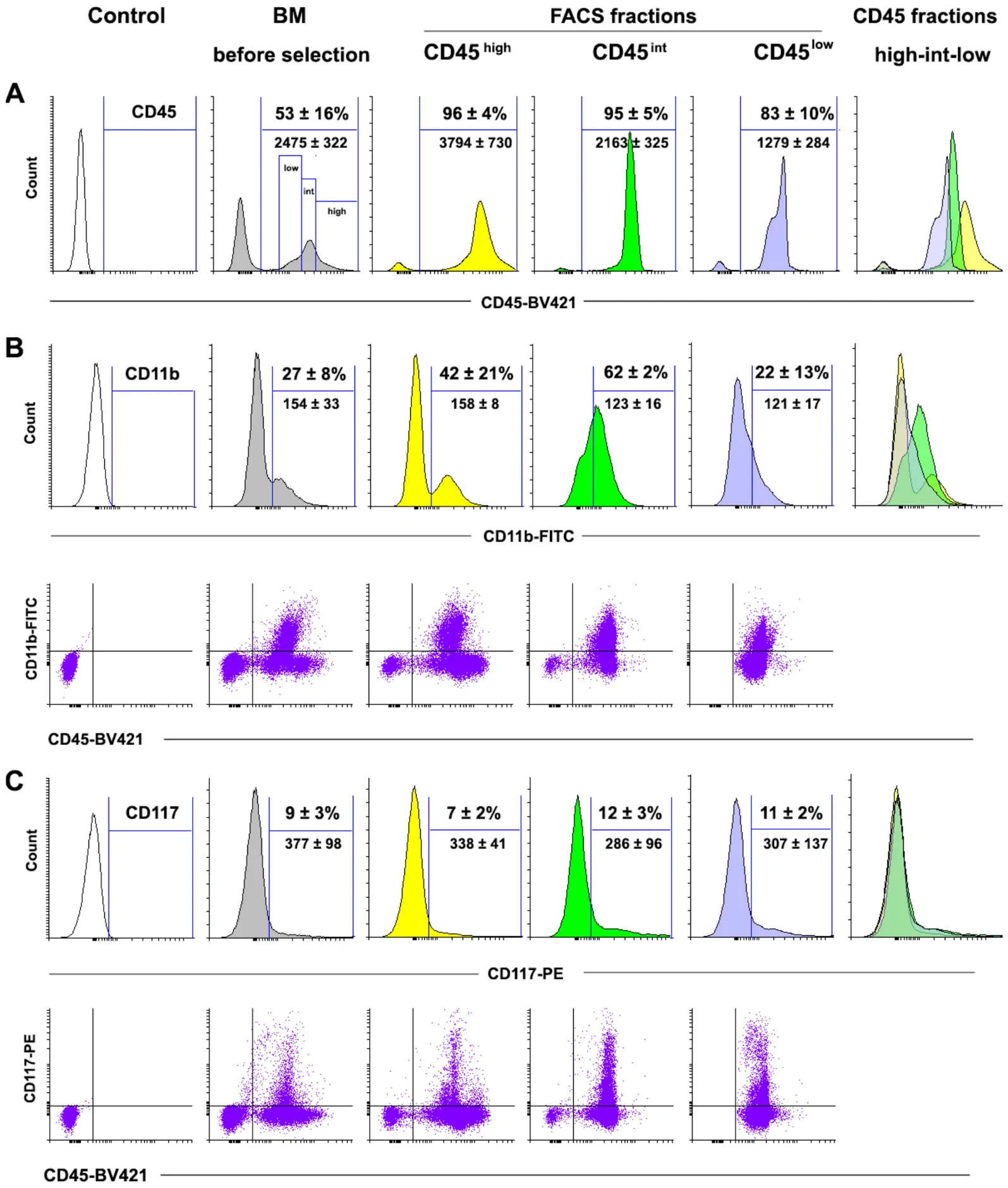
Fluorescent selection of 3 fractions of CD45+ VM cells. (**A**) Gates for fractionation of CD45+ BM cells and flow cytometry analysis of CD45-positive FACS-selected populations. (**B**) CD11b-FITC and CD11b-FITC/CD45-BV421 profiles of selected fractions. (**C**) CD117-PE and CD117-PE/CD45-BV421 profiles of selected fractions. The cytometric profiles are shown as one-dimensional histograms, as well as two-dimensional histograms vs. CD45. Data represent mean values ± SD for three independent experiments.

To check validity of findings obtained with MACS-MEL procedure, we performed FACS selection of CD45-positive cells from BM. BM cells were labeled with a mixture of CD45-BV421, CD117-PE and CD11b-FITC antibodies and sorted using a BD FACSAriaIII Cell Sorter. Figure 7A shows the gating of labeled BM cells for CD45^low^, CD45^int^, and CD45^high^ fractions that were subsequently selected. In general, the histograms of the selected CD45^low^, CD45^int^, and CD45^high^ cells in MACS-MEL and FACS were generally similar (Figure 5A and Figure 7A). Cytometric analysis of the selected cells showed that the proportion of CD45+ cells in the CD45^in^t and CD45^high^ cell fractions was at least 95%, and the MFI of CD45^high^ cells was 1.75 and 3.0 times higher than that of CD45^int^ and CD45^low^ cells, respectively (Figure 7A, Table 7). The proportion of CD117+ cells was minimal (about 7%) in the CD45^high^ fraction, whereas in the cells of the CD45^low^ and CD45^int^ fractions, the proportion of CD117+ cells was 1.6 times higher (Figure 7C), which agrees with the results obtained by MACS-MEL approach (Figure 5F). CD11b was present in all three cell fractions selected in FACS (Figure 7B) and MACS-MEL (Figure 5C), while in both cases the maximum proportion of CD11b cells was in CD45^int^ cell fractions (Figure 5C and Figure 7B).

CFU frequency of the BM cell was 183 ± 29 CFU/10^5^ cells, which was comparable to CFU numbers for BM in experiments with MACS-MEL. However, in FACS fractions, CFU frequencies dropped quite significantly and were at least an order of magnitude lower than in BM under same conditions, representing a drastic difference to MACS-MEL fractions. One might note, however, that the CFU in the CD45^high^ fraction was 2–3 times lower than in the CD45^low^ and CD45^int^ fractions (Table 8), in general agreement with what was observed with MACS-MEL selection.

## 4. Discussion

Fluorescence-activated cell sorting (FACS) and magnetic cell sorting are currently the most widely used technologies for cell separation. FACS is unrivalled in terms of resolving power and other features and is commonly used for basic research in small animal models. Magnetic cell sorting is attractive due to its simplicity and accessibility, but its major strength lies in its ability to process large quantities of cells. This feature determines its two major application areas, namely, the preparation of selected cell fractions for downstream analyses and separations, and large-scale isolation of specific cell subsets for various human cell therapy protocols. Currently, cell preparations for human transplantation are obtained using magnetic sorting and not FACS, as this permits us to obtain required numbers of selected cells in short time and avoid cell damage [23,24,25]. Magnetic sorting is approved by FDA for CD34+ cell isolation [26].

MACS-MEL, a method for selecting cells with high or low expression of a surface marker, is also based on magnetic sorting. MACS-MEL exploits the fact that when cells are labeled with a small amount of magnetic particles, only cells with a high level of expression of this marker will acquire sufficient magnetization and remain on the column, while cells with an intermediate and low expression level will end up in the flow-through fraction. This latter fraction, in turn, can be further fractionated using an increased number of magnetic particles.

Despite the successful demonstration of the MACS-MEL method using a model system of retrovirally transduced NIH 3T3 cells [3], it was not clear whether it was also applicable to real-life cases of natural primary cell systems. Taking into account an ever-increasing need for mass selection and transplantation of BM cells [27,28,29], in this work, we validated MACS-MEL approach on BM cells, demonstrating that MACS-MEL can be a useful tool for cell regenerative therapy. The choice of CD45 in the current work as a selection marker was stipulated by its commonality on BM cells, significant variability of expression depending on cell type and differentiation status, and considerations of its use as a therapeutic target in a number of studies [30].

The basic protocol including two selection rounds convincingly demonstrated the efficiency of MACS-MEL for selection of cell populations differing both in expression of the selected marker and in other properties including functional ones. We also tested an extended protocol with three rounds of selection to analyze in detail the separation potential of the method and the properties of the entire spectrum of selected populations. The combined results of two protocols indicate that consecutive column 1 to 3 fractions contain cell subsets with progressively diminishing CD45 expression, from CD45^high^ subset to CD45^low^ subset, respectively. Importantly, the decrease in CD45 expression is accompanied by reduced expression of myeloid differentiation markers Gr-1 and CD11b, which are important proteins playing a role in the pathogenesis of various diseases [31,32], as well as B-lymphoid marker CD19 [33].

In contrast to differentiation markers mentioned above, column 1 to 3 fractions demonstrated a clear elevation of expression of the key regulator of stem/progenitor cells, CD117, with the proportion of CD117^+^ cells increasing in various experiments from about 3–8% to 10–20% in column 1 and 2 fractions, while in the column 3 fraction of the extended experiment, CD117^+^ cells were even more abundant. In our extended protocol, the CFU frequency of the selected CD45^low^ and CD45^int^ subsets was 16 and 7.5-fold higher than in the CD45^high^ subset, respectively, which correlated well with the differences in the frequency of CD117^+^ cells in the populations. The obtained results reveal clearly that both the expression of CD117 and CFU activity of CD45-positive cells increase in parallel with the decrease in CD45 expression. These data thus are in good agreement with those reported for the human system [12,13].

To additionally validate findings obtained with MACS-MEL procedure, we performed fluorescence-mediated sorting of CD45+ BM cells into CD45^high^, CD45^int^ and CD45^low^ fractions. The results of the FACS fractionation were generally similar to those obtained with MACS-MEL protocol. A comparison of CD11b in the fractions of CD45+ cells in FACS and MACS-MEL showed that in both cases the maximum proportion of CD11b cells was in the fractions CD45^int^. In addition, the proportion of CD117+ cells was lower in the FACS CD45^high^ fraction than in the CD45^int^ and CD45^low^ fractions, which is similar to the proportion of CD117 cells in respective MACS-MEL fractions.

Although MACS-MEL and FACS methods demonstrated general similarity in cell surface phenotypes of selected fraction, the CFU analysis revealed, rather unexpectedly, quite a different picture. In particular, CFU potential of cells in selected FACS fractions dropped at least by an order of magnitude as compared to BM in the same experiment. In MACS-MEL separations, in contrast, only the CD45^high^ fraction had several-fold lower CFU potential, while both CD45^int^ and CD45^low^ fractions demonstrated substantially higher CFU frequencies compared to BM (Figure 4A and Figure 6A). It should be noted that several-fold reduction in colony-forming potential was reported earlier in human hematopoietic system for CD34-positive FACS-selected fractions as compared to MACS fractions [34]. Although the exact reasons for the drastically reduced CFU output after FACS selection are currently unclear, it appears that a factor known in the literature as the sorter induced cell stress (SICS), which imposes significant mechanical and oxidative stress on sorted cells, may be a main driving factor in this phenomenon. SICS was observed after FACS selection in astrocytes [35], Jurkat и NIH/3T3 cell lines [36] and myeloid cells [37]. SICS is presumably one of the factors limiting application of FACS for cell therapy. In particular, De Geyter et al. [38] noted serious health problems in several children whose parents’ spermatozoa were FACS selected for intra-cytoplasmic sperm injection.

Finally, an important issue that deserves discussion is potential limitations of the approach. In our work, we used two-stage MACS-MEL selection in a basic protocol, and three-stage selection in an extended one. The reduced CD45-normalized CFU frequency in fractions column 3 and flow-through 3 as compared to flow-through 2 suggest that the longer treatment times and/or extra stress of the 3^d^ selection stage might be a cause of the observed CFU frequency drop. To test these assumptions, we conducted additional tests aimed at elucidation of these concerns and described in detail in Supplementary Materials. The obtained results demonstrate that extended processing times in MACS-MEL protocol have little effect on cell state and CFU potential. The results also point to the absence of gross negative effects of repeated column passages on cell state and CFU frequency. Nevertheless, certain changes in cytometric profiles were observed in the 2nd column run, which can possibly be explained by reduced resistance of selected cell subsets to mechanical stress during magnetic selection. However, this potential issue is likely to exist for the column-based magnetic selection approaches in general.

Thus, the main drawback specific to the MACS-MEL procedure seems to be a decreased cell yield. Our findings thus suggest that three selection rounds are likely a practical limit of this approach due to cumulative cell losses augmenting with each selection round. We estimate that for most cases of mass isolation of cell subsets, two selection rounds will suffice, representing an optimal compromise between resolution of target cell subsets and their yield. Although the number of required rounds depends on the objectives of the selection process, in certain cases and under appropriate selection conditions, the optimal results can possibly be obtained even with a single-round selection scheme. Of relevance, isolation of partially CD45-negative cells using AutoMACS Pro Separator in one selection step was recently reported for the rabbit system, although the enrichment of hematopoietic progenitors in isolated fractions, in our opinion, has not been conclusively demonstrated [39]. It should also be noted that, due to a limited resolving power of magnetic selection, successful application of MACS-MEL approach requires a fairly broad distribution of expression levels of selectable marker.

Summarizing the above, we believe that MACS-MEL can potentially be used to create automated processes for the production of cellular drugs currently under development [40].

## 5. Conclusions

The MACS-MEL method has previously demonstrated good selection efficiency in the model system of NIH 3T3-transduced cells. In the present study, its effectiveness in the selection of natural cell populations was confirmed for CD45-positive cells from BM. We believe that MACS-MEL is able to take its place among the technologies of mass cell separation for the selection of target cells and for the primary enrichment of populations with target cells.

## Figures and Tables

**Table 1:** Pairwise comparison of percentage of positive cells for BM and selected fractions (see Figure 2). The *p*-values were calculated using two-tailed Student’s *t*-test. CF indicates column fraction. *p* values with *p* ≤ 0.05 are in bold.

Variants	CF 2 µL MBs vs. BM	CF 8 µL MBs vs. BM	CF 2 µL MBs vs. CF 8 µL MBs
CD45	**9.4 × 10^−5^**	**2.8 × 10^−4^**	0.497
CD11b	**1.3 × 10^−4^**	**3.2 × 10^−4^**	0.052
Gr-1	**1.8 × 10^−4^**	**2.1 × 10^−3^**	0.467
CD117	**7.2 × 10^−3^**	0.174	**4.3 × 10^−3^**
CD115	0.128	0.535	0.540

**Table 2:** MFIs of cells of BM and selected fractions. Results represent mean ± SD of 4 separate experiments. CF indicates column fraction.

	BM	CF 2 µL MBs	CF 8 µL MBs
CD45	268.92 ± 27.89	356.85 ± 71.19	254.72 ± 35.36
Gr-1	563.44 ± 99.98	696.26 ± 147.28	506.67 ± 206.72
CD11b	567.85 ± 195.10	916.75 ± 297.15	628.01 ± 237.89
CD117	353.78 ± 83.64	222.76 ± 37.89	223.54 ± 65.27
CD115	295.58 ± 274.61	303.12 ± 300.13	300.13 ± 251

**Table 3:** Pairwise comparison of MFIs for BM and selected fractions. The *p*-values were calculated using two-tailed Student’s *t*-test. CF indicates column fraction. *p* values with *p* ≤ 0.05 are in bold.

	CF 2 µL MBs vs. BM	CF 8 µL MBs vs. BM	CF 2 µL MBs CF 8 µL MBs
CD45	0.076	0.416	**0** **.** **013**
CD11b	**0** **.** **032**	0.144	**0.022**
Gr-1	**0.037**	0.466	0.079
CD117	**0.047**	**0.039**	0.982
CD115	0.662	0.173	0.209

**Table 4:** CFU frequencies per 10^5^ cells in BM and isolated cell fractions. CF indicates column fraction. Data represent 4 independent experiments. Shown are mean values ± SD.

CFU Type	BM	CF 2 µL MBs	CF 8 µL MBs
Total CFUs	233.3 ± 62.5	55.8 ± 29.7	386.9 ± 187.3
CFU-GEMM	58.2 ± 42.1	4.3 ± 4.7	105.6 ± 76.6
CFU-GM	104.7 ± 58.2	30.8 ± 28.7	216.1 ± 148.9
BFU-E	70.4 ± 39.2	20.7 ±9.0	65.3 ± 31.9

**Table 5:** Pairwise comparison of total CFUs and specific CFU types in BM and isolated cell fractions. The *p*-values were calculated using two-tailed Student’s *t*-test. CF indicates column fraction. *p* values with *p* ≤ 0.05 are in bold.

Pairwise Comparison	*p* Values			
	Total CFUs	CFU-GEMM	CFU-GM	BFU-E
CF 2 µL MBs vs. CF 8 µL MBs	**4.3 × 10^−6^**	**1.5 × 10^−4^**	**3.4 × 10^−4^**	**1.2 × 10^−4^**
BM vs. CF 2 µL MBs	**9.8 × 10^−9^**	**2.2 × 10^−4^**	**6.9 × 10^−4^**	**3 × 10^−4^**
BM vs. CF 8 µL MBs	**1.3 × 10^−2^**	0.074	**2.5 × 10^−2^**	0.728

**Table 6:** Cell yield in the extended experiment, in particular yield of total cells in the column-retained and flow-through fractions, as well as the proportion of mechanical cell loss after selection stages. Absolute cell yield is the yield of cells in a given fraction relative to the starting cell number of the entire experiment (25 × 10^6^). The relative cell yield is the proportion of cells in the fractions in a given selection stage relative to the number of cells at the start of this stage.

Stage	Fraction	Total Cell Yield	
		Absolute Cell Yield	Relative Cell Yield
BM	Starting Cell Number	100% (2.5 × 10^7^ Cells)	
Stage 1	Column	14.32%	14.32%
	Flow-through	62.80%	62.80%
	Mechanical loss	22.88%	22.88%
Stage 2	Column	20.10%	32.01%
	Flow-through	30.66%	48.82%
	Mechanical loss	12.04%	19.17%
Stage 3	Column	2.76%	9.02%
	Flow-through	24.08%	78.54%
	Mechanical loss	3.82%	12.44%

**Table 7:** Pairwise comparison of CD45 MFIs for BM and selected FACS CD45 fractions (see Figure 7). The *p*-values were calculated using two-tailed Student’s *t*-test. *p* values with *p* ≤ 0.05 are in bold.

Variants	CD45^low^	CD45^int^	CD45^high^
BM	**0.008**	0.303	0.207
CD45^high^	**0.011**	0.162	-/-
CD45^int^	**0.024**	-/-	-/-

**Table 8:** CFU frequencies of BM and FACS cell fractions per 10^5^ cells. Data represent 3 replicates. Shown are mean values ± SD.

	BM	CD45^high^	CD45^int^	CD45^low^
Total CFUs	183 ± 29	6.6 ± 5.8	17.8 ± 19.2	13.4 ± 22

## Data Availability

Original data are available upon request.

## Supplementary Materials

The following supporting information can be downloaded at: https://www.mdpi.com/article/10.3390/cells15171517/s1, Figure S1: Flow cytometry gating strategy; Figure S2: Schemes of analysis of effects of extended incubation of BM cells (A) and column-selected combined fractions (B) on their CFU potential; Figure S3: Scheme of analysis of effects of repeated passages of CD45-positive BM cells through columns on their CFU potential; Figure S4: Cytometry analysis of CD45 cells after selection on columns 1, 2 and 3; Table S1: CFU frequencies per 10^5^ BM cells after prolonged incubation in PBE medium; Table S2: CFU frequencies per 10^5^ cells of combined fractions 1 and 2 after prolonged incubation in PBE medium; Table S3: CFU frequencies per 10^5^ cells for column 1 and combined 2 and 3 fractions. References [41,42,43,44] are cited in the supplementary materials.
