# Supplementary material for: Magnetic-Assisted Fractionation of Bone Marrow Cells into Subsets Differing in CD45 Expression Levels, Surface Phenotypes and Functional Properties
Source: Cells. 2026 Aug 23;15(17):1517. doi: 10.3390/cells15171517 (PMC13565698; doi:10.3390/cells15171517)
Supplement: Supplementary file 1 [file cells-15-01517-s001.zip › cells-4265101-supplementary.pdf]

Supplementary Materials

Flow cytometry gating strategy

Bone marrow is highly heterogeneous and consists of complex combination of various cell populations differing significantly in marker expression, size, and granularity. Therefore, logarithmic scales for FSC and SSC were used to visualize the entire BM cell population. For more accurate positioning of BM cell sizes on the FSC logarithmic scale, we used calibration particles of different sizes. Figure S1 shows the distribution of BM cells on the logarithmic scale of FSC-A and SSC-A, taking into account the particle sizes. Figure S1A shows a population of BM cells ranging in size from 6 μm to 10 μm. According to literature data, the size of erythrocytes in murine blood ranges from 4-7 μm [41,42], and cells of this size have also been found in mouse BM [43,44], which provides a basis for classifying these cells as cells of the erythroid series of BM. We stained the BM cells with TER-119 antibodies, a marker of erythroid cells. Figure S1D shows that cells from 6 to 10 μm in size are stained with these antibodies and belong to the cells of the erythroid series of BM. Fig. S1B and Fig. S1C show the sequential gating of the BM population, where the gates FSC-A/SSC-A and FSC-H/FSC-A exclude debris and cell clusters, respectively. The cells of magnetically selected fractions were gated in the same way. In general, cytometric analysis of BM cells and cells of selected fractions included at least 90% and 85% of events, respectively. The results of cytometry of colored and unstained cell fractions were presented in the form of histograms and dot plots. Gates on histograms and dot plots were established using unstained cells. The MFI values and the proportion of positive cells in the populations were calculated using histograms and the Flowing Software program.

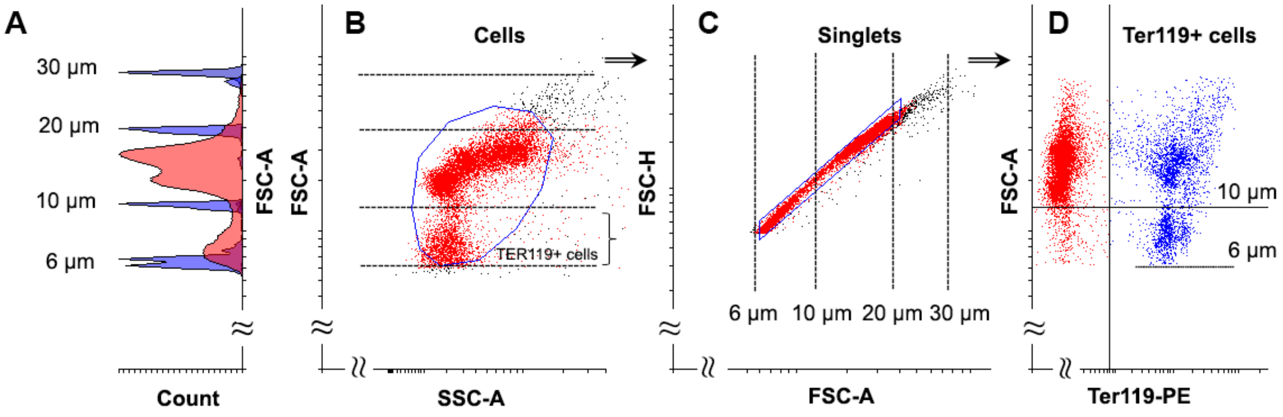

Figure. S1. Flow cytometry gating strategy. A) Histogram of BM cells and the position of calibration particles on the FSC-A logarithmic scale, B) Dot plot FSC-A/SSC-A of BM cells, lines indicate the size of calibration particles, C) Dot plot FSC-H/FSC-A of BM cells, lines indicate the size of calibration particles, D) Dot plot FSC-A/Ter119-PE for BM cells stained with Ter119-PE antibodies.

Analysis of factors affecting performance of MACS-MEL procedure

The extended 3-stage selection experiment described in the main text suggested existence of potential negative effects of the MACS-MEL procedure on cell state and CFU potential. One of the nearly invariant features of MACS-MEL procedure is the extended time of cell processing due to repeated selection cycles. We therefore first tested how the prolonged incubation of BM cells or column-selected cells in PBE isolation medium (shortly after BM isolation compared to 6-8 hrs and 30 hrs after BM isolation) may affect their viability and CFU potential. Fig. S2 depicts scheme of this experiment. The obtained results (Tables S1 and S2) demonstrate that prolonged incubations, even as lengthy as 30 hrs, do not substantially affect the studied parameters.

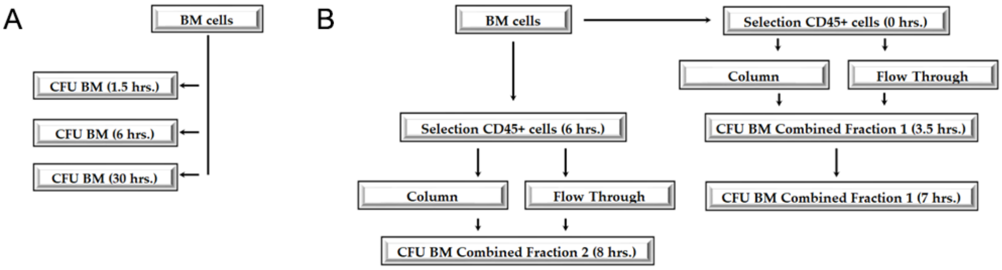

Figure S2. Schemes of analysis of effects of extended incubation of BM cells (**A**) and column-selected combined fractions (**B**) on their CFU potential.

Table S1. CFU frequencies per 10^5 BM cells after prolonged incubation in PBE medium. Results represent mean ± SD values for one experiment, with measurements performed in triplicate. The p-values were calculated using One Way ANOVA test.

	**BM (1.5 hrs)**	**BM (6 hrs)**	**BM (30 hrs)**	**p**
CFU	194.44 ± 12.73	147.22 ± 9.62	183.33 ± 80.36	0.485

Table S2. CFU frequencies per 10^5 cells of combined fractions 1 and 2 after prolonged incubation in PBE medium. Results represent mean ± SD values for one experiment, with measurements performed in triplicate. The p-values were calculated using One Way ANOVA test. CF indicates combined fraction.

	**CF 1 (3.5 hrs)**	**CF 1 (7 hrs)**	**CF 2 (8 hrs)**	**p**
CFU	280.56 ± 100.46	308.33 ± 52,04	288.89 ± 29,27	0.877

We next asked whether repeated column isolation cycles may affect the profiles, viability and CFU potential of cells. To this end, we first isolated CD45-positive cells from BM using 10 μl of MBs according to a basic protocol. This fraction was the subjected to two cycles of column isolation, each time combining the isolated column fraction and flow-through fraction to exclude the losses of cells due to incomplete cell binding to column. These two combined fractions column 2 and 3 were compared to the 1st isolated fraction. The Fig. S3 depicts the scheme of this experiment, while the Fig. S4 presents the results of this experiment

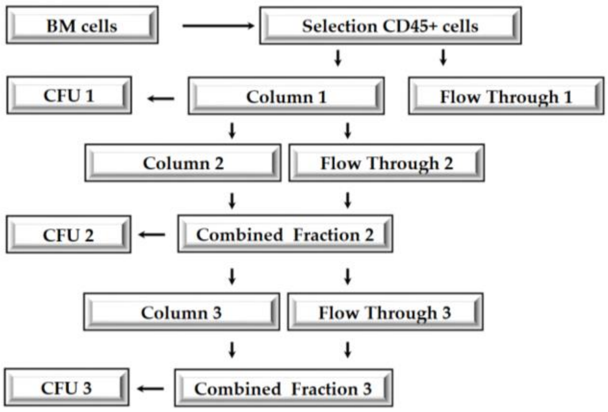

Figure S3. Scheme of analysis of effects of repeated passages of CD45-positive BM cells through columns on their CFU potential.

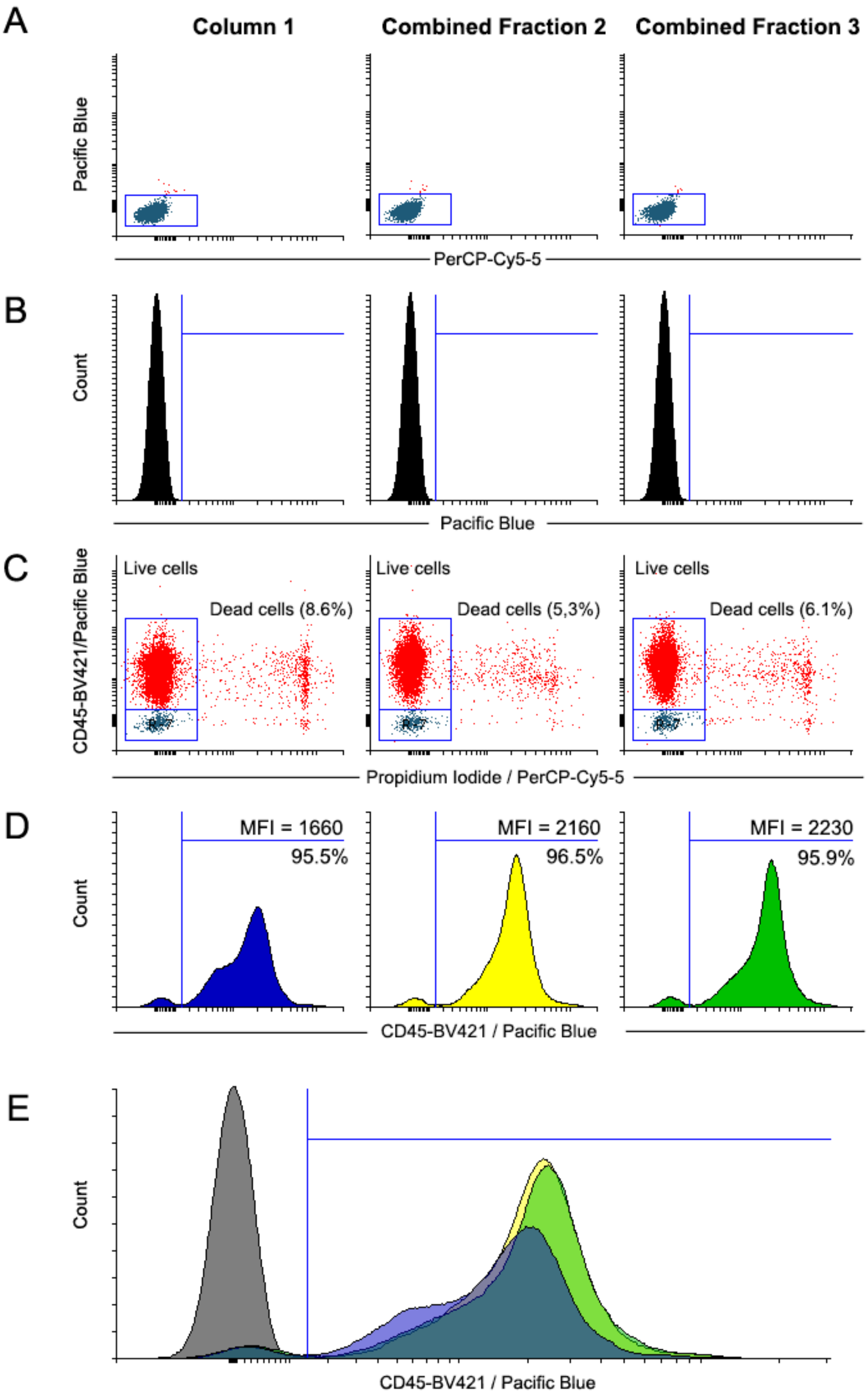

Figure S4. Cytometry analysis of CD45 cells after selection on columns 1, 2 and 3. A, B) Dot plots and histograms of unstained cells. C, D) Dot plots and histograms of cells stained with CD45-Brilliant Violet 421 and propidium iodide, E) Combined histograms of cells stained with CD45-Brilliant Violet 421.

The cytometric profiles demonstrate that the proportion of the CD45+ cells with lower CD45 expression levels was reduced in combined fractions 2 and 3 as compared to the column 1. In accordance with our experiments described in the main text that demonstrated the increased CFU frequency for cells with reduced CD45 expression, the CFU frequencies in column 2 and 3 fractions were somewhat lower compared to the column 1 fraction (Table S3). However, this reduction in CFU frequency was not statistically significant. Importantly, the cytometric

profiles, viability and CFU frequencies were quite similar for column 2 and 3 fractions. Thus, the obtained results point to the absence of gross negative effects of extended processing times and repeated column passages on CFU frequency, but also suggest a possibility of reduced representation of specific cell subsets, probably due selective loss as a result of reduced resistance to mechanical stress.

Table S3. CFU frequencies per 10^5 cells for column 1 and combined 2 and 3 fractions. Results represent mean ± SD values for one experiment, with measurements performed in triplicate. The p-values were calculated using One Way ANOVA test.

	**CFU 1**	**CFU 2**	**CFU 3**	**p**
CFU	194.44 ± 100.4	125 ± 54.64	141.67 ± 36.32	0.484
